# Assessing nutrition-related knowledge, attitudes, and practices towards breast cancer prevention among female students at the Federal University of Oye-Ekiti, Nigeria

**DOI:** 10.1186/s43046-024-00226-2

**Published:** 2024-06-03

**Authors:** Ibiwumi Damaris Kolawole, Oni Kunle, Kayode Ajayi, Thomas Prates Ong

**Affiliations:** 1 Department of Food and Experimental Nutrition, University of Sao Paulo, Sao Paulo, Brazil; 2 Department of Food Science and Technology, Federal University of Oye-Ekiti, Oye, Nigeria; 3 Department of Human Nutrition and Dietetics, Afe Babalola University, Ado Ekiti, Nigeria

**Keywords:** Breast cancer, Nutrition knowledge, Attitudes, Prevention, Nigeria

## Abstract

**Background:**

Breast cancer remains a complex disease and leading cause of cancer-related death in Nigerian women. Recently, the role of nutrition has been highlighted in the etiology of breast cancer.

**Methods:**

The aim of this research was to evaluate the nutrition-related knowledge, attitude, and practices of female university students. We also investigated the correlation between their demographic characteristics and their knowledge and attitudes of the survey participants. A descriptive cross-sectional study was carried out among female students at the Federal University of Oye (FUOYE), Nigeria. Participants completed self-administered questionnaires designed to assess their knowledge, attitude, and practices concerning cancer prevention. Statistical analysis was performed using SPSS 20, and significance was set at *p* < 0.05.

**Results:**

Out of the 402 students who received the questionnaire, 300 completed it. The average age of the participants was 21.26 years with a standard deviation of 2.68. There was generally limited knowledge regarding breast cancer risk factors, with 45% of participants citing family history as the most recognized risk factor. Overall, knowledge level was influenced by the participants’ permanent place of residence and course of study. Attitudes towards the impact of maternal and paternal nutrition on breast cancer prevention were notably low. Additionally, less than half of the participants demonstrated good dietary practices.

**Conclusion:**

This study revealed low levels of nutrition-related knowledge concerning cancer prevention, accompanied by poor dietary habits among the participants. These results suggest a possible link between inadequate knowledge about breast cancer prevention and the observed poor dietary practices among the participants. The frequent consumption of unhealthy foods among the participants may be a pointer to higher risk of breast cancer in the future, emphasizing a need for health education targeted at this group.

## Background

Breast cancer continues to be a substantial cause of cancer-related death and the most frequently diagnosed cancer among women irrespective of the racial and ethnic factors [1]. Prevention has been identified as the most cost-effective approach to control the increasing mortality and morbidity rates caused by breast cancer [2]. The disease impact varies widely across the populations, and African women including Nigerians are diagnosed at an earlier age and tend to display a more aggressive tumor phenotype [3]. Although developing countries present three times lower incidence of breast cancer compared to developed countries, the mortality to incidence rates is higher in the former due to disparities in access to adequate information, screening, diagnostic, and adequate treatment services [4]. In Nigeria, breast cancer accounts for 26% of all cancer cases with 70% women presenting at an advanced stage of the disease, and this makes an effective treatment more challenging [5, 6]. Studies have reported that most Nigerian women know little or nothing about symptoms and risk factors of breast cancer [7, 8]. Many known modifiable risk factors in prevention of breast cancer such as those related to diet and lifestyle have only been widely studied in the developed countries but not in developing countries including Nigeria.

Most countries in sub-Saharan Africa including Nigeria are focusing on treating infectious diseases such as HIV/AIDS, and tuberculosis, and noninfectious diseases such as breast cancers, are given low priority leading to low awareness and treatment facilities of breast cancer in the country [5, 9]. The incidence of breast cancer continues to rise especially among young Nigerian women, and the reason for the increasing incidence is not fully known, although this may be due in part to the lack of understanding of the local risk factors. Conceivably, poor understanding of the underlying risk factors that increase the mortality and mortality rates of the disease among Nigerian population could also contribute to increasing incidence of breast cancer in the country.

Our present study was conducted to fill in the research gap regarding the perspective of Nigerian women on nutrition-related breast cancer risk factors towards prevention of the disease. Adequate understanding of these factors could significantly contribute to research and enhance breast cancer prevention. Further, this study could potentially increase awareness and promote healthy lifestyles and reduce the incidence of the disease in Nigeria.

## Methodology

### Study design and population

Female participants (ages 17–30 years) were randomly selected from all departments within the main campuses of the Federal University of Oye-Ekiti. A written informed consent was obtained from each participant before collection of data. The data were collected using a self-administered questionnaire designed according to Lahiji et al. [20] with some modification to adapt to the local context and meet up with the objectives of study. An initial pilot study was conducted using 10 students to ensure clarity of questions and wording errors prior to the definitive study. Content validity was assessed quantitatively and qualitatively to ensure data accuracy.

The final questionnaire consisted of four sections: (A) sociodemographic, (B) knowledge (8 questions), (C) attitude (10 questions), and (D) dietary practices (31 food items). The responses were anonymous to ensure confidentiality.

The inclusion criteria for the study included those of 17–30 years old and had not been diagnosed with breast cancer. After oral and written explanation to the participants about the aim and objectives of the study, an informed consent was obtained from the participants before administering the questionnaires.

The self-administered questionnaires were used to collect sociodemographic characteristics information, nutrition-related knowledge, attitudes, and eating pattern towards breast cancer prevention.

### Demographic questionnaire

Each participant was asked to report their age, marital status, religion, ethnicity, faculty of study, level of education, family history of breast cancer, and current and permanent place of residence.

### Knowledge questionnaire

There were eight nutrition-related knowledge questions (related to breast cancer prevention), and their respective responses were scored. Score of 1 was given to the right answers and score 0 to the wrong answers. The overall scores of knowledge for individual range from 0 to 8.

### Attitude questionnaire

Attitude questions were evaluated on a 5-point Likert scale in which 1 point was given to strongly disagree, 2 points to disagree, 3 points to agree, 4 points to strongly agree, and 0 for I do not know.

### Practice questionnaire

A food frequency questionnaire (FFQ) was used to assess dietary practice. The FFQ was compiled based on the commonly consumed foods in the southwest region of Nigeria according to the West African food composition table [11]. The modified FFQ included nine food groups: fish, meat, and their products, fruits and vegetables, snacks, cereal and grains, fat and oil, beverages, and alcoholic drinks. Participant indicated the frequency of consuming these foods in the past 3 weeks, with response options including daily, 2–3 times a week, once a week, and occasionally. Participants reported how frequent they consumed these foods during the past 3 weeks.

### Participant’s recruitments

A descriptive cross-sectional study was conducted to evaluate the level of nutrition-related knowledge, attitude, and practices of female undergraduate students at the Federal University of Oye Ekiti, Nigeria. The study was carried out between October 2022 and February 2023 in Nigeria. Students from the faculty of basic sciences, agricultural sciences, arts, management science, social sciences, and education participated in this study. Only 300 out of 402 participants completed the questionnaires resulting to a response of 74.6%.

### Statistical analysis

The obtained data were coded and analyzed using SPSS 20 software. The variables were determined using frequency, percentage, mean, and standard deviation. The relationship between categorical data variables was evaluated using chi-square test. Linear regression analysis was performed to predict the level of knowledge and attitude. The significance level in all the tests was set at 0.05.

## Results

Responses were gotten from total of 300 out of 402 female students that participated in this study. The sociodemographic profiles of the participants are presented in Table 1. The mean and standard deviation of the age of the participants was 21.26 ± 2.68. Most of the students (93.3%) were single, most were Christian (85%), and majority of them (85.7%) were from Yoruba ethnic group.

**Table 1:** Frequency distribution of sociodemographic profiles of the students (*N* = 300)

Variables	(%)
Age	
≤ 23	246 (82%)
≥ 24	54 (18%)
Mean age	21.26 ± 2.68
Marital status	
Single	286 (95.3%)
Married	10 (3.3%)
Others	4 (1.3%)
Religious	
Christian	255 (85%)
Muslim	45 (15%)
Ethnicity	
Yoruba	257 (85.7%)
Igbo	19 (6.3%)
Hausa	4 (1.3%)
Others	20 (6.7%)
Faculty	
Basic sciences	119 (39.7%)
Agricultural sciences	86 (28.6%)
Arts	58 (19.3%)
Management science	17 (5.6%)
Social sciences	10 (3.3%)
Education	10 (3.3)
Year/level of study	
First year	47 (15.7%)
Second year	82 (27.3%)
Third year	123 (41%)
Fourth year	48 (16%)
Current place of residence	
Student hostel	46 (15.3%)
Rented apartment	237 (79%)
With family	17 (5.7%)
Permanent residence	
Rural	99 (33%)
Urban	201 (67%)
History of BC	
First degree	9 (3%)
Other degree	7 (2.3%)
No family history	284 (94.7%)

### Knowledge

The nutrition-related knowledge question on breast cancer prevention and the percentage in proportion of the participants that answered correctly are shown in Table 2. Family history identified by 45% of the participants was the most identified risk factor for breast cancer development. It is also necessary to note that only 24% of this participant identified both obesity and physical inactivity as the risk of breast cancer. The result obtained from this study showed that less than 30% of the participants had overall good knowledge on breast cancer risk factors. More than 70% of the students did not know “age,” “being female,” “alcohol consumption,” “early menstruation,” “physical inactivity,” “obesity,” “breastfeeding,” and “oral contraceptive” as breast cancer risk factors. Although, 85% and 83% of the students respectively knew that breast cancer can developed without family history and the disease is not contagious but majority of them lack adequate knowledge on breast cancer risk factors. A total of 73% of the participants knew breast cancer does not only affect postmenopausal women, while more than 80% knew breast cancer can be prevented and majority knew diagnosis of breast cancer do not always lead to death.

**Table 2:** Knowledge questions related to breast cancer prevention of participants (*N* = 300)

Knowledge question	Correct response (%)
Is breast cancer the most common cancer in Nigerian women?	248 (82.7%)
Is –-a determined risk factor for breast cancer?	
a. Age	63 (21%)
b. Being female	85 (28%)
c. Smoking	91 (30%)
d. Alcohol consumption	83 (28%)
f. Unhealthy diet	100 (33%)
g. Physical inactivity	71 (24%)
h. Obesity	72 (24%)
i. Family history	135 (45%)
j. Breastfeeding	68 (23%)
k. Use of oral contraceptive	77 (26%)
l. Early menstruation	51 (17%)
Knowledge of the food that can modify the risk of breast cancer?	120 (40%)
Is breast cancer preventable?	268 (89.3%)
Is breast cancer contagious?	251 (83.7%)
Does diagnosis of breast cancer always lead to death?	193(64.3%)
Does breast cancer only affect postmenopausal women?	220 (73.3%)
Can breast cancer be developed without family history?	256 (85.3%)

The relationship between demographic characteristics and good (scores equal to mean or more) and poor (scores lesser than mean value) level of nutrition-related knowledge towards breast cancer prevention among the participants is presented in Table 3. Also, Table 4 indicated the results of correlation analysis between demographic characteristics and knowledge.

**Table 3:** Relationship between demographic characteristics and nutrition-related knowledge (*N* = 300)

Characteristics	Good	Poor	*p*-value
Age			
≤ 23	134 (44.7%)	112 (37.3%)	0.37
≥ 24	33 (11%)	21 (7%)	
Marital status			
Single	162 (54%)	124 (41.3%)	0.24
Married	3 (1%)	7 (2.3%)	
Others	2 (0.7)	2 (0.7%)	
Religion			
Christian	145 (48.3%)	110 (36.7%)	0.32
Muslim	22 (7.4%)	23 (7.6%)	
Ethnicity			
Yoruba	142 (47.3%)	115 (38.3%)	0.54
Igbo	12 (4%)	7 (2.3%)	
Hausa	1 (0.3%)	3 (1%)	
Others	12 (4%)	8 (2.7%)	
Faculty			
Basic sciences	71 (23.7%)	48 (16%)	**0.03**
Agriculture	51 (17%)	35 (11.7%)	
Arts	31 (10.3%)	27 (9%)	
Management science	8 (2.6%)	9 (3%)	
Social sciences	5 (1.6%)	5 (1.6%)	
Education	1 (0.3%)	9 (3%)	
Year/level of study			
First year	26 (8.7%)	21 (7%)	< **0.00001**
Second year	67 (22.3%)	15 (5%)	
Third year	48 (16%)	75 (25%)	
Fourth year	26 (8.7%)	22 (7.3%)	
Current place of residence			
Student hostel	18 (6%)	28 (9.3%)	**0.01**
Rented apartment	142 (47.3%)	95 (31.7%)	
With family	7 (2.3%)	10 (3.3%)	
Permanent residence			
Rural	51 (17%)	48 (16%)	0.30
Urban	116 (38.7%)	85 (28.3%)	
History of BC			
First degree	7 (2.3%)	2 (0.7%)	0.32216
Other degree	3 (1%)	4 (1.3%)	
No family history	157 (52.3%)	127 (42.3%)	

^*^Chi-square test. Significant *P*-value (< 0.05) was bolded

**Table 4:** Linear regression model of total knowledge score (*N* = 300)

Variables	*R*^2^	SE	B	*P*
Age	0.001	0.034	0.026	0.441
Year of study^a^	0.011	0.0008	0.001	0.064
History of BC	0.00001	0.236	0.016	0.945
Faculty^b^	0.026	0.062	0.177	**0.005**

^a^Categorized as years 1, 2, 3, and 4

^b^Categorized as basic sciences, agricultural sciences, management sciences, social sciences, education, and arts. Significant *P*-value (< 0.05) was bolded

### Attitude

The frequency and percentage in proportion of the participants with adequate attitudes towards breast cancer prevention are shown in Table 5. The mean attitude of the participants was 23.14. In this study, 84% and 79.4% of the participants agreed that consumption of fruits and vegetables can reduce the risk of breast cancer, while 64% and 79.7% of them had a negative attitude towards the influence of maternal and paternal nutrition in breast cancer development on offspring during preconception and pregnancy. However, 85% and 65% of the participants believed that consumption of healthy diets and maintaining healthy weight can lower the risk of breast cancer respectively. A total of 34% of the students did not know about the protective effect of breastfeeding against breast cancer, and only 74% and 59% respectively agreed that avoiding cigarette smoking and limiting consumption of alcohol are protective against breast cancer development. The linear regression model of total attitude score is indicated in Table 6.

**Table 5:** Nutrition attitude questions related to breast cancer prevention (*N* = 300)

Question	Right response
Do you believe regular consumption of vegetables can reduce the risk of breast cancer?	238 (79.4%)
Do you believe regular consumption of fruit can reduce the risk of breast cancer?	252 (84%)
Do you believe breastfeeding can reduce the risk of breast cancer?	199 (66.3%)
Do you believe that consumption of a healthy diet can reduce the risk of breast cancer?	265 (85%)
Do you believe avoidance of smoking can reduce the risk of breast cancer?	224 (74.4%)
Do you believe that maternal nutrition during pregnancy can increase the daughter’s risk of breast cancer in the future?	108 (36%)
Do you believe that paternal nutrition during preconception can increase breast cancer in the future?	61 (20.3%)
Do you believe that limiting alcohol consumption can reduce the risk of breast cancer?	177 (59%)
Do you believe regular physical exercise can reduce the risk of breast cancer?	221 (73.7%)
Do you believe maintaining a healthy weight can reduce the risk of breast cancer?	195 (65%)

**Table 6:** Linear regression model of total attitude score (*N* = 300)

Variables	*R*^2^	SE	B	*P*
Age	0.000004	0.113	0.012	0.910
Year of study^a^	0.026	0.002	0.007	**0.005**
History of BC	0.001	0.769	0.447	0.56
Faculty^b^	0.0006	0.206	0.091	0.658

^a^Categorized as years 1, 2, 3, and 4

^b^Categorized as basic sciences, agricultural sciences, management sciences, social sciences, education, and arts. Significant *P*-value (< 0.05) was bolded

### Practice

The intake pattern from the food group indicated that the most daily consumed foods and food ingredients in this study was rice, snacks, processed foods, fruits, palm oil, sugar, and garri. Less than half of the participants had a good dietary practices. The consumption pattern showed that 22% of the participants daily consumed fish, and 46% consumed it 2–3 times weekly. The consumption of homemade food was 67% daily. The intake of processed foods was 30% daily, and 41% of them consumed it 2–3 times weekly. Approximately, 30% of the participants consumed fruit daily. Among 79% of students that showed positive attitude towards regular consumption of vegetable in breast cancer prevention, only 21% of them ate vegetables daily, and 39% consumed it more twice weekly. We also found that less than 8% of the students consumed legumes, unprocessed grains, and red meat daily, while less 10% of them consumed this more three times weekly. Only 3% of the participants drank alcoholic drinks daily, and about 6% took it 2–3 times weekly. A total of 30% of the participants consumed carbonated drink 2–3 times weekly.

Although, married participants with first degree history of breast cancer and living with family had better nutritional practices towards breast cancer prevention compared to others but the difference is not significant.

## Discussion

This study assessed the nutrition-related knowledge, attitude, and practices towards breast cancer prevention among the female undergraduate students at the Federal University of Oye Ekiti, Nigeria. There are reports on knowledge, attitude, and practice on breast cancer screening methods and treatment in Nigeria [12–16], but to the best of our knowledge, no research has been done on nutrition-related knowledge, attitude, and practice towards prevention of breast cancer in Nigeria. Therefore, this is the first study reporting this among female university students in Nigeria. The purpose for this study is the need to have information on nutrition-related knowledge, attitudes, and practices towards prevention of breast cancer.

This study showed that most of the participants were aware that breast cancer is the most common cancer among Nigerian women but had inadequate knowledge on the risk factors involved in breast cancer development and on foods that can modify the risk factors of the disease. Family history, unhealthy diet, smoking, being female, and alcohol consumption were the mainly identified risk factors of the malignancy by the participants. These results indicated lack of awareness of risk factors involved in breast cancer among Nigerian women. Similar findings were reported in their respective studies [17, 18]. Only 45% of the participants indicated that family history can increase the risk of breast cancer. This result is similar to the one reported by Akhigbe and Omuemu in a study carried out among Nigerian female health workers towards their knowledge on breast cancer screening methods [12]. Despite majority of the participants knew breast cancer could be prevented, less than 30% of them had good knowledge on the risk factors. Age, an important risk factor for developing breast cancer was only indicated by 21% of the participants in this study. This is consistent with a report by Tazhibi and Feizi in a population-based study on awareness level of breast cancer risk factors in Iranian women [19]. Linear regression model showed that the level of knowledge in breast cancer prevention might be affected by course of study, consistent with what was reported in a study [20]. Besides, 74% of them had overall good knowledge towards breast cancer prevention except for the risk factors. Additionally, better level of nutrition-related knowledge was recorded among those living in urban areas compared to rural areas, and this might be due to increasing levels of cancer awareness in the urban area as this result is similar to what was reported by Azubuike and Okwuokei [13].

Our study showed that majority of the participants had poor attitude towards the influence of maternal and paternal nutrition during preconception and pregnancy in breast cancer prevention. Most of the participants showed good attitudes towards the role of regular consumption of fruits and vegetables in the disease prevention, but the best attitude was seen in regular consumption of healthy diet and fruits (Tables 5 and 7). Again, the level of nutrition-related attitudes towards breast cancer prevention was more positive among participants at the faculty of basic sciences, students at the second year of study, and those living permanently in the urban area.

**Table 7:** Relationship between attitude and demographic characteristics (*N* = 300)

Characteristics	Positive	Negative	*p*-value
Age			
≤ 23	128 (42.7%)	118 (39.3%)	0.26
≥ 24	32 (10.7%)	21 (7%)	
Marital status			
Single	152 (50.6%)	134 (44.7%)	0.70
Married	8 (2.7%)	2 (0.6%)	
Others	1 (0.3%)	3 (1%)	
Religion			
Christian	139 (46.3%)	116 (38.7%)	0.48
Muslim	22 (7.3%)	23 (7.7%)	
Ethnicity			
Yoruba	136 (45.3%)	121 (40.3%)	0.68
Igbo	10 (3.3%)	9 (3%)	
Hausa	4 (1.3%)	0 (0%)	
Others	11 (3.7%)	9 (3%)	
Faculty			
Basic sciences	76 (25.3%)	43 (14.3%)	**0.05**
Agriculture	38 (12.7%)	48 (16%)	
Arts	30 (10%)	28 (9.3%)	
Management science	8 (2.7%)	9 (3%)	
Social sciences	4 (1.3%)	6 (2%)	
Education	5 (1.7%)	5 (1.7%)	
Year/level of study			
First year	17 (5.7%)	30 (10%)	**0.00004**
Second year	61 (20.3%)	21 (7%)	
Third year	57 (19%)	66 (22%)	
Fourth year	26 (8.7%)	22 (7.3%)	
Current place of residence			
Student hostel	23 (7.7%)	23 (7.7%)	0.80
Rented apartment	128 (42.7%)	109 (36.3%)	
With family	10 (3.3%)	7 (2.3%)	
Permanent residence			
Rural	47 (15.7%)	52 (17.3%)	0.13
Urban	114 (38%)	87 (29%)	
History of BC			
First degree	5 (1.7%)	4 (1.3%)	0.84
Other degree	3 (1%)	4 (1.3%)	
No family history	153 (51%)	131 (43.7%)	

With regard to dietary practices, the most frequently consumed foods in this study are processed foods (such as noodles, pasta, pizza), white bread, polished rice, fish, snacks (pastry), fried foods, carbonated drinks, sugar, and palm oil. The World Cancer Research Fund (WCRF) recommended everyone to embrace a healthy lifestyle such as being physically active; consumption of a diet loaded with fruits, vegetables, and legumes, and lesser consumption of processed foods high in sugar and fat; and intake of carbonated drinks [2]. In this study, only 30% and 21% daily consumed fruits and vegetables respectively. This is similar to the reports that Nigerian university students infrequently consumed fruits and vegetables [10, 21, 22]. The low consumption of fruits and vegetables in this study may be due to inadequate nutritional knowledge, food unavailability, and pricing [23, 24]. Fruits and vegetables are more expensive than some processed foods and not adequately available on university campuses possibly because of their low self-life and low demand. In relation to the protective effect of fish in breast cancer, 68% of the participants consumed fish frequently. Higher intake of fish has been reported to be associated with lower risk of breast cancer [25]. Our findings on high consumption of processed foods are consistent with a study by Oguizu and Celestine among adolescents in Nigeria on consumption of ultra-processed foods: 74.7% consumed carbonated drinks, 59.9% consumed instant noodles, and 68.8% consumed white bread frequently [26]. Apparently, Nigeria white bread contains potassium bromate used by bakers at a concentration between 3.6 µg/g and 12.16 µg/g to enhance bread texture and volume against 0.02 µg/g recommended by United States Food and Drug Agency [27, 28]. Similarly with a previous study that reported high consumption of snacks among adolescents in Nigeria [29], we found that 39% of the participants consumed snacks (pastry) daily, and 27.6% ate it 2–3 times in a week. This finding is also consistent with the result obtained from a study among university undergraduate students indicating that most students consumed pastry snacks daily [30]. Although, in the last two decades, there has been a significant shift in dietary patterns from normal traditional diets to ready-to-eat foods including snacks due to urbanization and technology explosion [31]. However, snacks are not good sources of dietary fiber and have been associated with diet-related noncommunicable diseases such as obesity. Otemuyiwa and Adewusi reported these foods to be calorie dense that can promote obesity among women [32]. The availability of many snack shops in the school premises and affordability of the snacks could be the reasons for its high consumption among the participants. Consumption of snacks and fried foods may increase the risk of breast cancer [33, 34]. Our study indicated that 57.7% and 55.66% consumed fried foods and sugar more than two times weekly respectively. This study showed that about 50% of the participants consumed palm oil daily. Palm oil has been reported to contains high amounts of saturated fats (50%) which facilitate body fat accumulation when consumed in excess and can be detrimental to human health [35]. Exposure to palm oil during gestation and lactation period results in changes of the offspring’s adipose tissue development and metabolism in adult life, increasing visceral fat deposition and subsequently influencing the risk of having metabolic diseases [36]. The consumption of legumes among the participants is very low in this  study, and less than 8% ate legumes more than twice weekly. This finding is consistent with a report by Akah et al. [11]. Legumes contain dietary fiber, amino acid, and polyphenols, antioxidants, and many other phytochemicals that can lower the risk of cancer and other cardiovascular diseases [37]. Therefore, higher consumption of fiber-rich foods may reduce the risk of estrogen receptor (ER −) and progesterone receptor (PR −) breast cancer [38]. In summary, the participants had poor dietary practices. The consumption of foods that has been reported to lower the risk of breast cancer was less satisfactory in this study. Although, students older than 24 years of age had better dietary practices compared to younger students, and a similar result was reported by Lahiji et al. towards breast cancer prevention [20].

The small size of the study population may not fully reflect the broader Nigerian population. There are plans in progress to broaden the selection of cohorts to encompass a more diverse geographical range, thereby ensuring a more comprehensive representation of the entire Nigerian populace and enhancing the reliability of data interpretation.

## Conclusion

In conclusion, our studies showed inadequate knowledge about risk factors involved in breast cancer development, and this reflected in the dietary practices of the participants. The frequent consumption of unhealthy foods among the participants may be a pointer to higher risk of breast cancer in the future, emphasizing a need for health education targeted at this group.

## Data Availability

The data sets used and analyzed in this study are available from the corresponding author upon reasonable request.

## Abbreviations

BC: Breast cancer
PR: Progesterone receptor
ER: Estrogen receptor
WCRF: World Cancer Research Fund
FFQ: Food frequency questionnaire
HIV: Human immunodeficiency virus
AIDS: Acquired immunodeficiency syndrome
